# Automated quality assurance of HDR brachytherapy: Evaluation of Dwell‐position accuracy and Dwell‐time linearity via image processing of radiochromic films

**DOI:** 10.1002/pro6.70040

**Published:** 2025-12-23

**Authors:** Qingqing Yuan, Suyan Bi, En Lou, YaJun Jia, Zhitao Dai

**Affiliations:** ^1^ National Cancer Center/National Clinical Research Center for Cancer/Cancer Hospital and Shenzhen Hospital Chinese Academy of Medical Sciences and Peking Union Medical College Shenzhen China; ^2^ School of Medical Sciences Universiti Sains Malaysia Kubang Kerian Kelantan Malaysia; ^3^ Department of Physics and Key Laboratory of Artificial Micro & Nano‐structures of Ministry of Education School of Physics and Technology Wuhan University Wuhan China

**Keywords:** High‐dose‐rate brachytherapy, Dwell‐position accuracy, Dwell‐time linearity, Radiochromic film, Image processing

## Abstract

**Purpose:**

To develop and validate a fully automated quality assurance method for high‐dose rate (HDR) brachytherapy, enabling the precise evaluation of source dwell‐position accuracy and dwell‐time linearity using radiochromic film analysis and advanced image processing algorithms.

**Methods:**

A novel analytical framework was established to verify HDR source positional precision and dwell‐time linearity. Radiochromic films were irradiated under predefined treatment plans. Image preprocessing involved grayscale conversion, median filtering, rotation correction, and absolute coordinate calibration. The centroid of each dwell position was determined using two‐dimensional Gaussian fitting. Temporal linearity analysis employed radial grayscale summation within the radii of 250–400 pixels. Linearity was quantified using the coefficient of determination (R^2^), with R^2^ approaching 1 indicating optimal performance.

**Results:**

Automated Gaussian fitting achieved sub‐millimeter positional accuracy (maximum error ≤ 0.6 mm), eliminating manual intervention. Rotation correction algorithms effectively mitigated positional errors caused by film misalignment. Comparative analysis with commercial software yielded nearly identical results, validating the reliability of the method. Dwell‐time analysis demonstrated excellent linearity (*R^2^≥*0.999) over summation radii ranging from 150 to 400 dpi.

**Conclusions:**

This study presents a robust and fully automated quality assurance (QA) solution for HDR brachytherapy that simultaneously verifies sub‐millimeter‐level dwell‐position accuracy and temporal linearity. The integration of radiochromic film dosimetry with algorithmic image processing eliminates manual intervention and standardizes QA workflows, representing a substantial advancement in treatment safety and efficiency.

## Graphical abstract

This study develops a method combining radiochromic film and image processing to assess HDR brachytherapy source accuracy and linearity, achieving high precision and efficiency

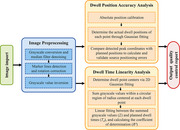

## 1 INTRODUCTION

Brachytherapy is a widely used radiotherapy modality for cancer treatment and typically employs a high‐dose‐rate (HDR) ^192^Ir source. This source is renowned for its rapid dose decay and high radiation intensity.^1^ HDR brachytherapy remains a cornerstone in treating malignancies such as cervical,^2^, ^3^ prostate,^4^, ^5^ and breast^6^ cancers owing to its inverse‐square law dose distribution, which enables precise tumor targeting while sparing normal tissues.

In cervical cancer brachytherapy, each session typically uses large doses of radiation (4‐8 Gy), with the peripheral organs at risks, particularly the small intestine, exhibiting low levels of radiation tolerance.^7^ Consequently, precise spatial control is critical during treatment delivery. Deviations in any of the three key physical parameters**—**source activity, dwell position, and dwell time**—**can substantially compromise tumor control probability or increase normal tissue complication rates, thereby impacting treatment efficacy. Currently, three‐dimensional (3D) image‐guided brachytherapy (IGBT), utilizing modalities such as computed tomography (CT) and magnetic resonance imaging (MRI), is widely adopted in clinical practice.^8^, ^9^ While two‐dimensional (2D) brachytherapy relies on X‐ray visible catheter markers for initial dwell‐position localization, 3D techniques typically employ CT‐visible fiducial markers within catheters, MRI line markers, or applicator‐based geometric offsets to define source positions. Therefore, routine quality assurance (QA) steps verifying source positioning accuracy are essential.^10^, ^11^

AAPM Task Group reports (TG40^12^ and TG56^1^) recommend the following key quality control (QC) standards for brachytherapy: (1) a source positioning tolerance of ±1 mm, (2) a timer accuracy tolerance of ±1%, and (3) source replacement either at the institution's discretion or on a quarterly basis. Commercial QA tools, such as well‐type ionization chamber, are widely employed for verifying source activity.^10^ However, beyond optional camera‐based systems, there is a lack of precise and efficient commercially available devices for the comprehensive testing of both source position and dwell‐time linearity.

Research into novel methods for source position and dwell‐time verification is ongoing. Watanabe et al. developed a pinhole imaging system for tracking the ^192^Ir source during HDR brachytherapy treatment and for inferring dwell times from integrated image pixel values.^13^ Otani et al. proposed a pretreatment verification method using a transparent applicator to evaluate channel length and dwell position.^14^ However, systematic differences may exist between delivery conditions in the transparent and actual applicators. Nose et al. reported a real‐time verification method using modified C‐ arm fluoroscopy.^15^ Although continuous source monitoring is ideal, its practicality may be restricted by concerns regarding additional radiation exposure to patients and staff, along with regulatory compliances (e.g., restrictions on operating multiple independent radiation emitters within a single treatment room). Tanderup *et al.* advocated for developing in vivo dosimetry into an effective independent verification tool to improve brachytherapy safety.^16^

Radiochromic films represent a gold standard in high‐resolution 2D radiation dosimetry owing their excellent spatial resolution and near tissue equivalence.^17^, ^18^, ^19^ Although the absence of reference markers on radiochromic films presents a challenge for the quantitative evaluation of source positions, these films remain clinically valuable for the qualitative verification of source location in brachytherapy.^11^, ^17^ To address the absence of reference positions, methods such as manually marking reference positions or incorporating tungsten wires have been proposed. This study utilized radiochromic films prepared using manually marked reference positions.^20^, ^21^ The purpose of this study was to develop an analytical method for assessing the precision of source positioning and the linearity of dwell times in HDR brachytherapy, utilizing radiochromic film dosimetry combined with image processing, and to implement an effective QA system.

## 2 MATERIALS AND METHODS

### 2.1 Measuring equipment and process

The following equipment and materials were utilized in this study.

**FIGURE 1:**
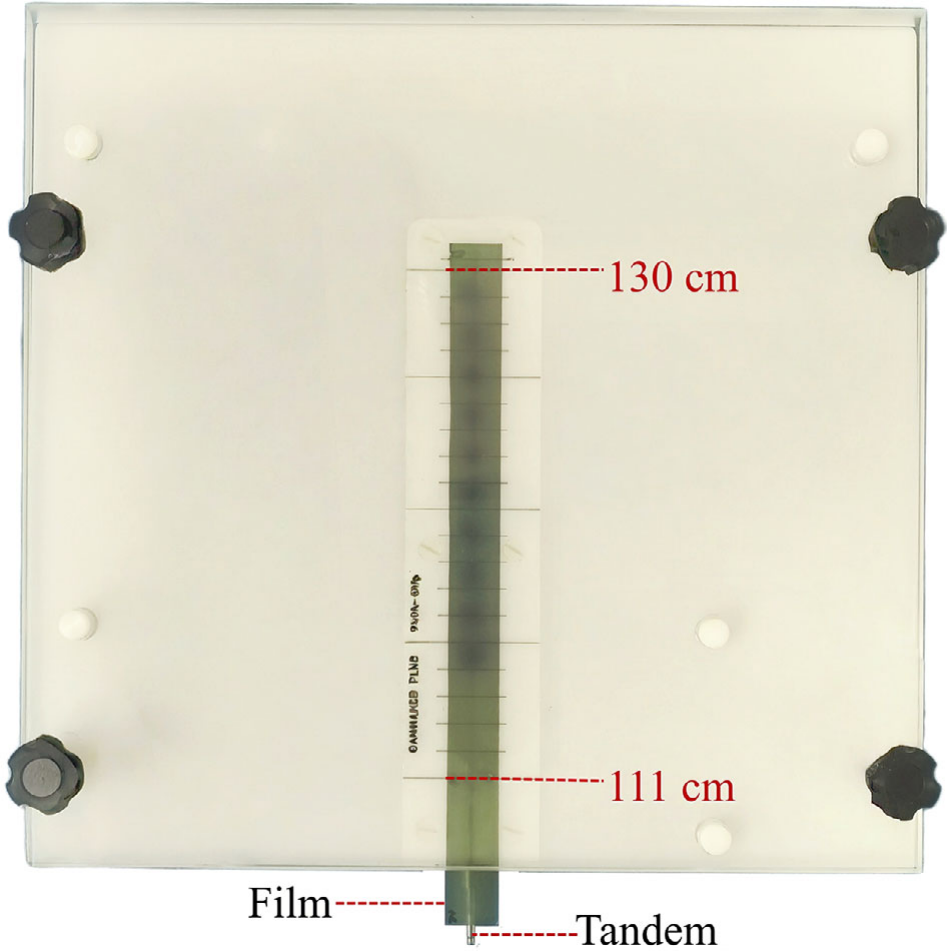
Schematic of PermaDoc Phantom and film positioning. The two longer red dashed lines correspond to the positions of the 130 cm and 119 cm scale lines on the PermaDoc Phantom, respectively.

1. **Brachytherapy System**: A Varian GammaMed Plus iX HDR brachytherapy system (Varian Medical Systems, Palo Alto, CA) was employed. The system utilized an ^1^⁹^2^Ir source with an activity of 3.7 TBq (with an activity uncertainty of ±5%, as per the calibration certificate). The source was calibrated using a well‐type ionization chamber (Model 3571, PTW, Freiburg, Germany) following AAPM TG 40 guidelines.^12^ Source transfer was performed using a 130‐cm stainless steel transfer tube.
2. **Measurement Phantom**: A PermaDoc Phantom (Catalog #9404‐GMP) was used for measurement. The device consisted of two matching 0.375‐in acrylic plates that allowed the insertion of a 8“×10” ready‐pack film. A radiochromic film was sandwiched between the acrylic plates, as shown in Figure 1.
3. **Radiochromic film**: The Gafchromic™ EBT3 radiochromic film (Ashland Inc., USA) was selected for its excellent tissue‐equivalent properties (effective atomic number Z = 7.35) and high spatial resolution (∼100 µm).^17^

As shown in Figure 1, before the measurement, two parallel lines separated by 19 cm were drawn on a pre‐cut EBT3 film (with approximate dimensions of 25 cm×4 cm) using a marker pen. A tandem was then inserted into the PermaDoc Phantom and the tube was connected properly. During the measurement, the bottom and top marked lines on the film were aligned with scale lines at 111 and 130 cm on the PermaDoc Phantom, respectively (as shown by the red dashed lines in Figure 1). Subsequently, the four fixing screws were tightened.

### 2.2 Film irradiation and digitization

Two simulated QA plans were developed using the Eclipse (Version 13.6) treatment planning system (Varian Medical Systems, Palo Alto, CA). To assess dwell‐position accuracy, measurements were obtained between 1300 mm and 1150 mm, with a total of 10 dwell points spaced 15 mm apart. At each point, the dwell time was set as 5 s to form clear and non‐overlapping exposure points on the film, and the measurement was repeated ten times. To evaluate the accuracy of dwell‐time linearity, four dwell points were set, with the distance between each dwell point being at least 15 mm. The dwell times at the four dwell points were set as 5, 10, 15, and 20 s, respectively. After exposure, the films were scanned using an Epson Expression 12000XL scanner with the following settings: 48‐bit full color, positive film mode, a scanning resolution of 300 dpi. The scanned images of the films were then saved in the TIFF format.

### 2.3 Image preprocessing

**FIGURE 2:**
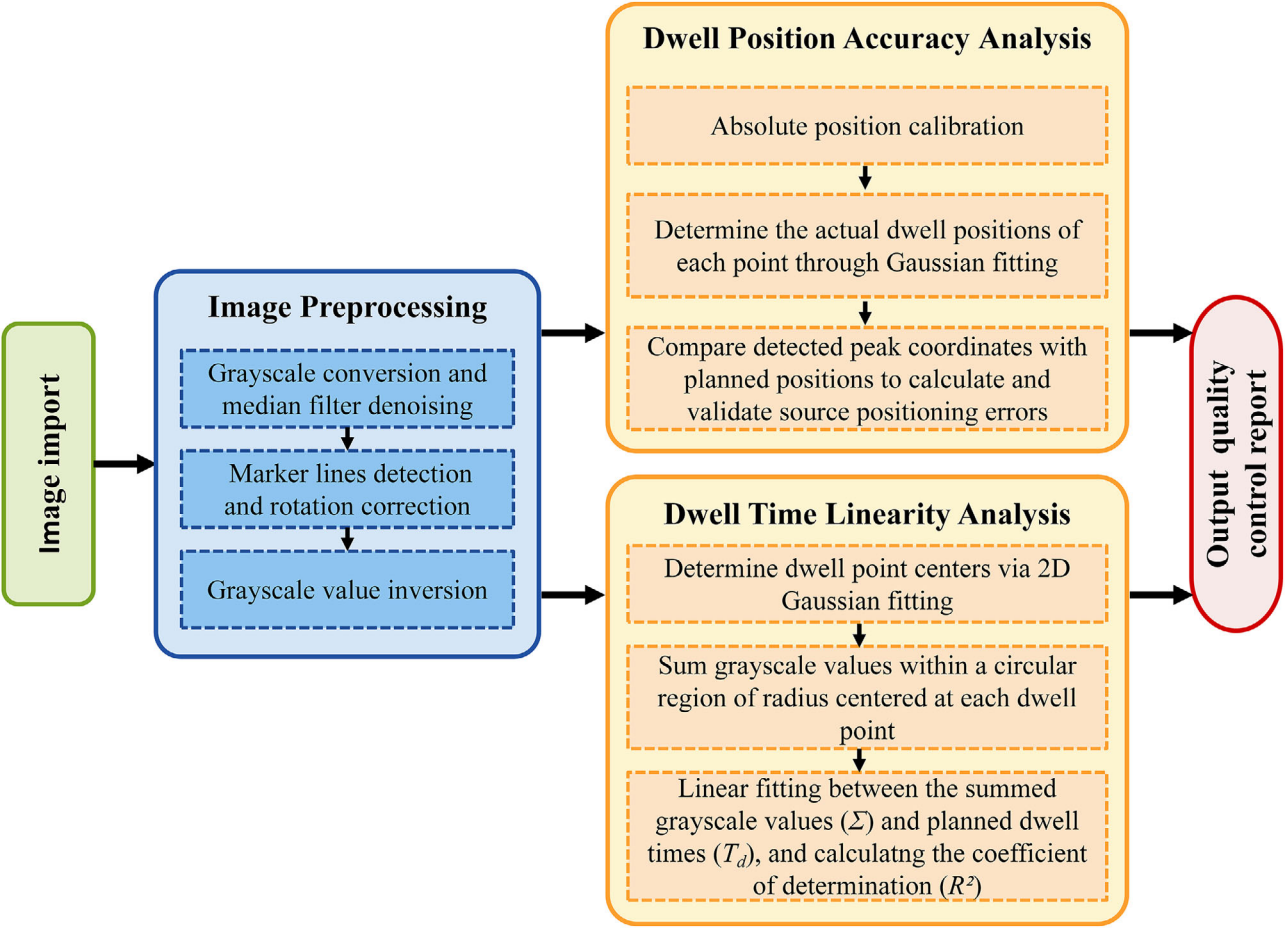
Flow chart of the analysis software.

**FIGURE 3:**
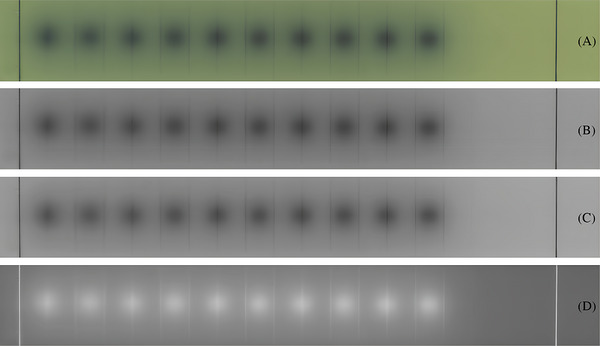
Example of the image preprocessing process. (a) is the original color image obtained by scanning; (b) is the image obtained after grayscale conversion and median filter denoising; (c) is the image after rotation correction; (d) is the image after grayscale inversion processing. The left and right lines in the figure are the marking lines at 130 cm and 111 cm, respectively.

A Brachytherapy QC software application was developed with MATLAB. Figure 2 shows a flow chart of the analysis software, which primarily includes three modules: image preprocessing, dwell‐ position accuracy analysis, and dwell‐time linearity analysis. Before conducting the analysis, the *imread[]* function in MATLAB was first used to import the scanned color images of the film in TIFF format into the software (as shown in Figure 3(a)). Subsequently, the images were preprocessed according to the following steps.

(1) **Grayscale conversion and median filter noise suppression**: The color images were converted to 8‐bit grayscale, and the grayscale image matrix *G(x,y)* was derived by processing the components of the three RGB channels using Formula (1):
(1) G(x,y)=0.299R+0.587G+0.114B

where R, G, and B represent the red, green, and blue components of color values, respectively, with their values ranging from 0 to 1.

A 3×3 median filter was selected to effectively reduce noise while preserving the small circular features of the dwell points for accurate localization:

(2) $$G_{median}\left(x,y\right)=median\left\{G\left(x,y\right)|{\left(x^{\prime }-x\right)}^{2}+{\left(y^{\prime }-y\right)}^{2}\leq 1\right\}$$

Where $G_{median}\left(x,y\right)$ is the filtered output. The neighborhood included all pixels within the Euclidean distance ≤1 (i.e., a 3×3 kernel). Figure 3(b) shows the image obtained after grayscale conversion and median‐filter‐based noise suppression.

(2) **Marker‐line detection and rotation correction**: The *graythresh()* function in MATLAB was used to automatically determine the global binarization threshold and subsequently binarize the denoised image obtained in step (1). The value of pixels with a grayscale value greater than or equal to this threshold was set to 255, whereas the value of those with a grayscale value less than the threshold was set to 0. Subsequently, the Hough transform (HT) algorithm was employed to automatically detect the marking lines in the binarized image. The straight lines formed by the detected points in the image domain were regarded as the marking lines. After obtaining the linear equations of the two marking lines, the angles between the lines and x axis (*θ_1_* and *θ_2_*) were calculated, and their average value θ was taken as the rotation angle of the image. Subsequently, rotation transformation was performed using Formula (3), where *(x', y')* denotes the coordinate pair obtained after the rotation transformation of the 2D coordinate pair *(x, y)*. Figure 3(c) shows the image obtained after rotation correction.
(3) $$\left(x^{\prime },y^{\prime }\right)=\left(x,y\right)\left(\begin{array}{cc} \cos\theta & -\sin\theta \\ \sin\theta & \cos\theta \end{array}\right)$$
(3) **Grayscale value inversion**: To facilitate subsequent Gaussian fitting and grayscale summation, an inverted grayscale matrix *g(x,y)* was obtained by applying grayscale inversion processing on the rotation‐corrected grayscale image obtained from step (2) using Formula (4). Figure 3(d) shows the image acquired after inversion processing.
(4) $$g\left(x,y\right)=1.0-G\left(x,y\right)$$

### 2.4 Dwell‐position accuracy analysis

The positional accuracy analysis module primarily performed the following steps.

**FIGURE 4:**
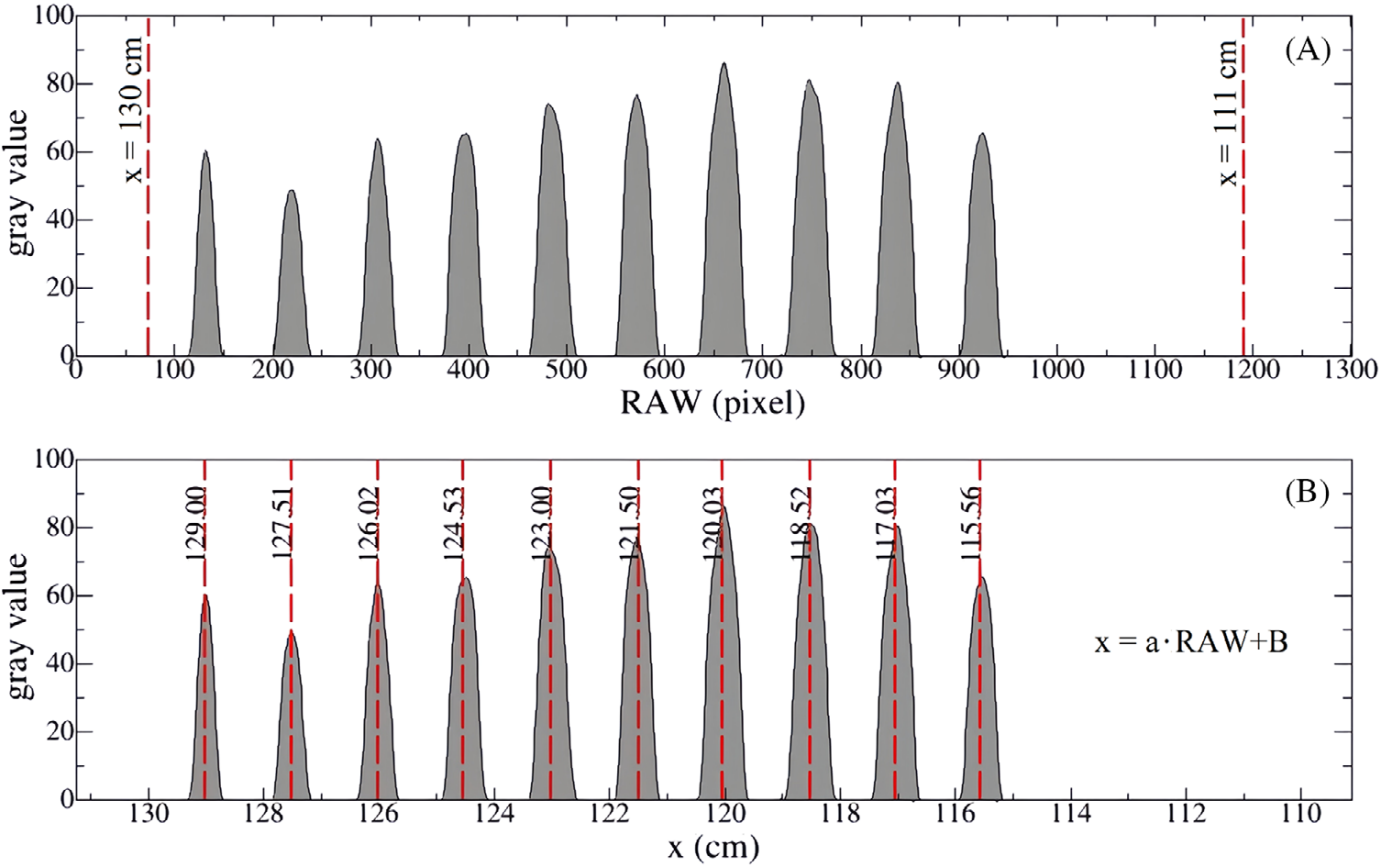
Example of dwell position analysis. (a) shows the original one‐dimensional data obtained by projecting the two‐dimensional image in Figure 3(d) onto the x‐axis. The two red dashed lines in the figure represent the absolute position coordinates at 130 cm and 111 cm, respectively. (b) presents the absolute coordinate data after absolute position calibration. Each dashed line in the figure, along with the corresponding number, indicates the position coordinate of each dwell point obtained through one‐dimensional Gaussian fitting.

**(1) Absolute position calibration**: To calculate dwell‐position error, absolute position calibration was performed on the image obtained after steps (1)–(3). As shown in Figure 4, RAW1 and RAW2 represent the horizontal coordinates in the pixel space corresponding to the two marking lines in the image (Figure 4(a)), with their absolute coordinates being 130 cm and 111 cm, respectively. Using the coordinates of the points (RAW1, 130) and (RAW2, 111), the formula for converting any pixel space coordinate RAW to the absolute position x can be obtained through linear fitting as follows:

(5) x=a·Raw+b

where a and b are the slope and intercept of the function, respectively. Using Formula (5), the spatial position of each pixel in the image obtained from step (3) was converted to the corresponding absolute position. Figure 4(b) shows the distribution of the absolute coordinates, as derived from the projection of the 2D image in the x‐direction.

**(2) Determination of the actual dwell positions corresponding to each point through Gaussian fitting**: Using Formula (6), Gaussian fitting was performed on each peak corresponding to the dwell points in the one‐dimensional projection image shown in Figure 4. As illustrated in Figure 4(b), the parameter μ derived from the Gaussian fitting of each peak was considered the actual position of the corresponding dwell point, which was denoted as x_meas_.
(6) $$f\left(x\right)=\frac{1}{\sqrt{2\pi \sigma }}\exp\left(-\frac{{\left(x-\mu \right)}^{2}}{2\sigma^{2}}\right)$$
**(3) Positional accuracy analysis**: The peak positions (*x_meas_*) obtained using Gaussian fitting for the 10 dwell points were compared with the coordinates of the 10 planned positions (*x_planned_*), as listed in Table 1. The positional error *Δx* for each dwell point was calculated as *Δx = x_meas._− x_planned_*. Additionally, a check was performed to determine whether the error fell within the error tolerance range of ±1 mm. For the actual positions and relative deviations of each dwell point derived from 10 repeated measurements, the mean value and standard deviation were calculated, respectively.
(4) **Comparison with RIT and robustness assessment**: To verify the reliability of our analytical method, the film images scanned after the 10 repeated measurements were imported into both the as‐developed HDR Brachytherapy QC software and RIT software (Radiological Imaging Technology, Inc., Colorado Springs, Colorado, USA)—a commercial software commonly used in daily clinical practice. The dwell‐position errors obtained from the two software packages were then compared. In addition, to validate the robustness of the software under different film scanning resolutions and the reliability of rotation error correction, each film was fitted at two resolutions: 300 dpi and 150 dpi. Furthermore, the 300‐dpi images were duplicated and rotated by 2° to simulate rotation errors introduced during manual scanning. Eventually, three groups of images with resolutions/rotation angles of 300 dpi/0°, 300 dpi/2°, and 150 dpi/0° were obtained. These three groups of images were imported into the HDR Brachytherapy QC software for analysis, and the corresponding dwell‐position error results were derived.

**TABLE 1:** Dwell‐position accuracy results of 10 dwell points (cm).

*x_planned_*	115.50	117.00	118.50	120.00	121.50	123.00	124.50	126.00	127.50	129.00
$\bar{x_{meas.}}$	115.52	117.02	118.51	120.00	121.50	122.99	124.50	125.99	127.49	128.98
$\bar{\Delta x}$	0.02	0.02	0.01	0.00	0.00	−0.01	0.00	−0.01	−0.01	−0.02
$\sigma_{\Delta x}$	0.03	0.04	0.04	0.03	0.04	0.04	0.05	0.04	0.04	0.05
$M_{\Delta x}$	0.02	0.00	0.01	0.00	−0.01	0.00	−0.02	−0.01	0.00	0.00

### 2.5 Dwell‐time linearity analysis

To assess dwell‐time linearity, four dwell points were set, with the distance between each dwell point being at least 15 mm. The dwell times at the four dwell points were 5, 10, 15, and 20 s, respectively. The dwell‐time linearity analysis is irrelevant to the absolute position of each dwell point. The analysis can be directly performed using images in the pixel coordinate space. Specifically, it included the following steps.

1. **Determine dwell point centers via 2D Gaussian fitting**: For the preprocessed images, the central coordinates  (*x_i_,y_i_*) of the four dwell points were determined via 2D Gaussian function fitting.
2. **Sum grayscale values within a circular region centered at each dwell point**: With (*x_i_,y_i_*) as the center, the grayscale values of each pixel within a circle of a certain radius (150–400 pixels) around each dwell point were summed to obtain the corresponding Σ_i_ value, as shown in Figure 6(a).
3. **Linear fitting between Σ and T_d_**: First, the summed grayscale values of the four positions corresponding to the dwell times of 5, 10, 15, and 20 s were normalized by dividing the *Σ_i_* value of each dwell point by the grayscale sum of the dwell point with the dwell time of 20 s, thereby confining the grayscale sum Σ_i_ of each dwell point to the range of 0–1. Subsequently, a graph was plotted with the dwell time *T_d_* and grayscale sum *Σ* of the four dwell points as the coordinates. The gray scale sum *Σ* and dwell time *T_d_* corresponding to each dwell position were linearly fitted using Formula 7 (as shown in Figure 6(b)).
(7) $$\Sigma =m\cdot T_{d}+n$$

where m and n were obtained using linear regression with T_d_ as the independent variables and *Σ* as the dependent variable. The coefficients of determination (*R^2^*) were obtained for the linear fitting. *R^2^* ranges from 0 to 1, with *R^2^* = 1 signifying a perfect linear relationship.

## 3 RESULTS

### 3.1 Image processing

Following acquisition, all radiochromic film images were preprocessed using a systematic workflow, which encompassed grayscale conversion, median filtering, rotation correction, and grayscale inversion. As illustrated in Figure 3, these procedures considerably enhanced image quality and promoted the distinct visualization of both exposure points and reference marker lines. Specifically, rotation correction using HT eliminated positional inconsistencies arising from misalignment during scanning, thereby ensuring the reliability of subsequent quantitative analyses.

### 3.2 Dwell‐position accuracy

The automated software successfully detected all dwell positions by applying one‐dimensional Gaussian fitting to the projected grayscale distribution (Figure 4). The calibration of absolute position on the basis of reference marker lines (at 130 cm and 111 cm) enabled accurate mapping from pixel space to real‐world coordinates. Table 1 summarizes the results of positional analyses for the 10 dwell points, as acquired over multiple repeated exposures. Across all positions, the mean absolute error (*Δx*) with respect to the planned locations was within ±0.06 cm, which surpassed the AAPM recommended tolerance of ±1 mm. The standard deviation of these measurements was consistently below 0.05 cm, indicating high repeatability. No systematic bias in localization accuracy was observed across the measurement range.

**FIGURE 5:**
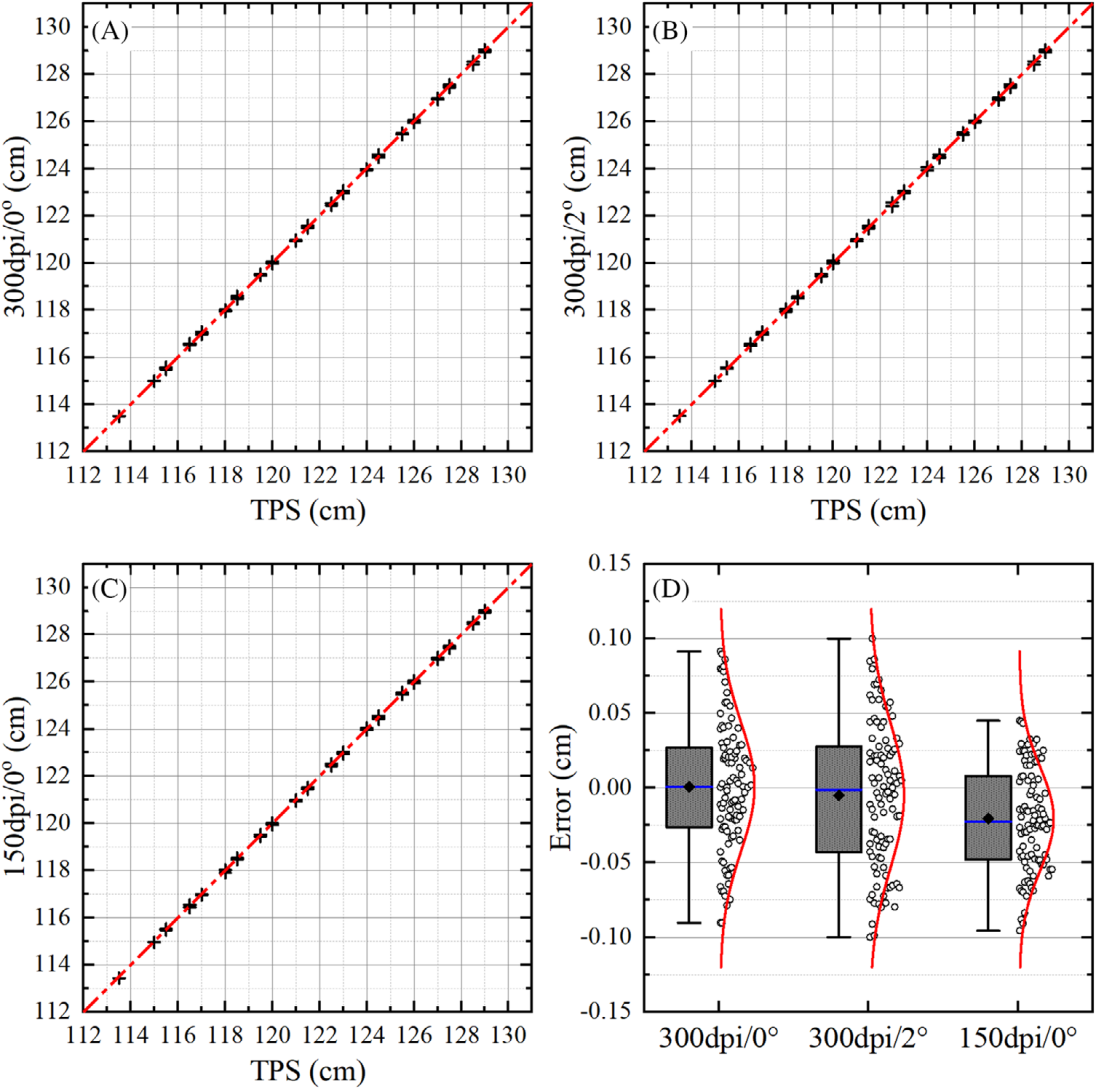
Comparison of position deviations of 10 dwell points obtained from 10 repeated measurements. In the figure, the gray color represents the analysis results of RIT software. The red, green, and blue colors correspond to the results under a scanning resolution and image rotation angle of 300 dpi/0°, 300 dpi/2°, and 150 dpi/0°, respectively. (a) shows the comparison of error corresponding to different dwell points, with error bars representing the standard deviation. (b) presents the comparison of boxplots of relative errors for all 10 dwell points.

To evaluate the robustness of the method, its performance was compared with that of a commercial software package (RIT) and tested under challenging conditions, including image rotation and reduced scanning resolution (Figure 5). The results showed that the positional error measurements derived by our algorithm were highly consistent with those from the RIT software. Crucially, artificially introducing a 2°rotation error (300 dpi/2°) or reducing the scanning resolution to 150 dpi did not substantially alter the accuracy, as shown by the overlapping error distributions in Figures 5a and 5b. This confirms the effectiveness of the rotation correction algorithm and the robustness of the overall analytical framework against common sources of procedural variance.

### 3.3 Dwell‐time linearity

**FIGURE 6:**
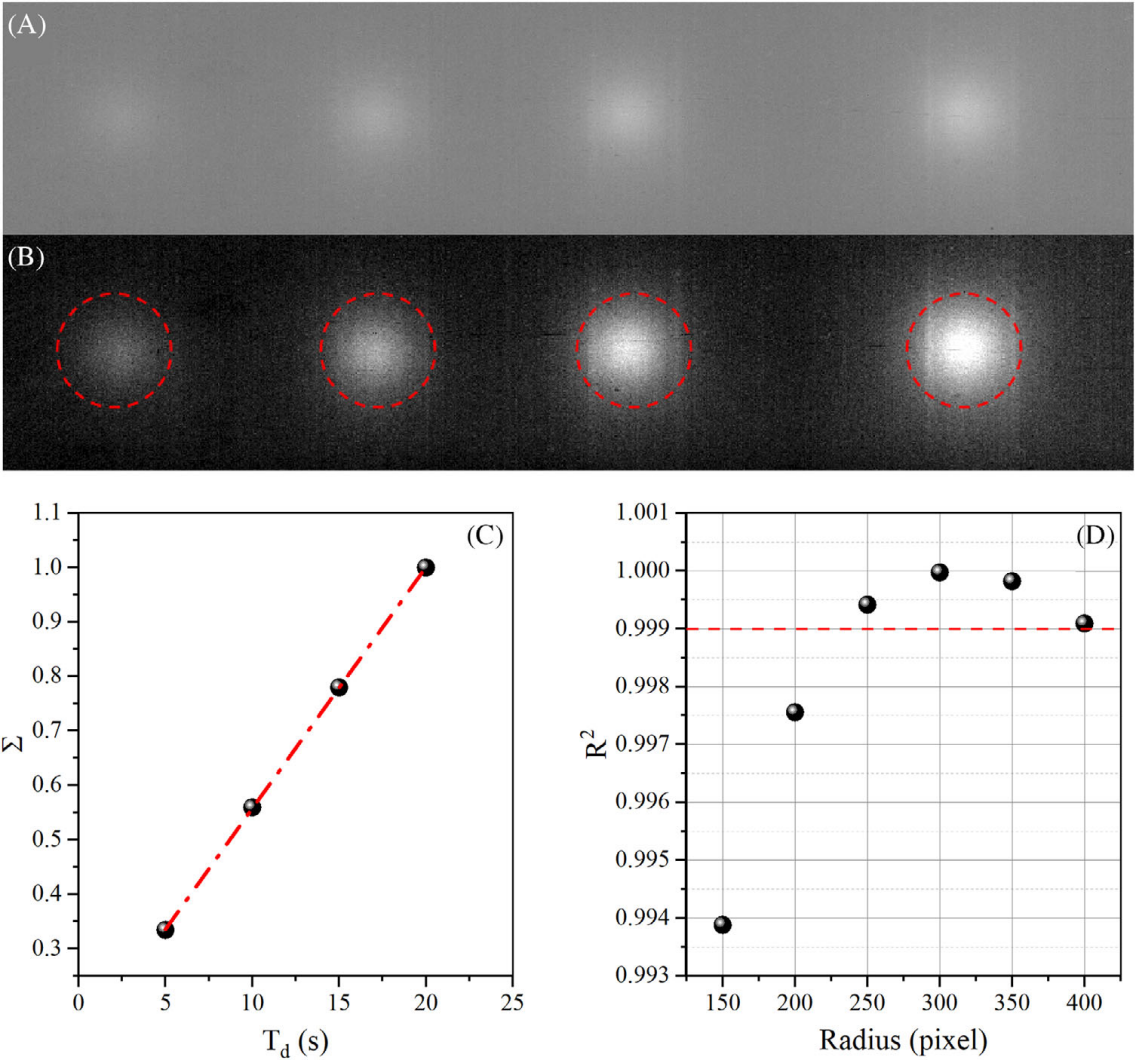
Illustration of dwell‐time linearity analysis. (a) is the image obtained after preprocessing. The red circles in the figures indicate the range used for grayscale sum calculation. (b)shows the linear fitting between the grayscale sum (Σ) and dwell time (T_d_) when the summation radius is 300 pixels. (c) presents the coefficient of determination for the linear fitting between the grayscale sum (Σ) and dwell time (T_d_) under different summation radii (150‐400 pixels).

To assess dwell‐time linearity, four dwell positions with varying programmed times (5, 10, 15, and 20 seconds) were analyzed. The grayscale sum within a circular region of interest (ROI) centered on each dwell point was normalized and fitted linearly to the preset dwell times. Figure 6(b) provides a detailed view of the linear fitting results when the summation radius was set to 300 pixels. The data points showed excellent alignment with the fitted line, indicating a robust linear relationship between the measured grayscale values and the programmed dwell times. The linear regression equation and corresponding statistical parameters confirmed the high quality of the linear fit.

The influence of summation radius on fitting quality was systematically investigated, as shown in Figure 6(c). The coefficient of determination (*R^2^*) values were calculated for summation radii ranging from 150 to 400 pixels. The *R^2^* values remained consistently high (>0.99) across the entire range, with optimal performance observed at intermediate radii. Specifically, the summation radius of 300 pixels yielded the highest *R^2^* value of 1.0, indicating nearly perfect linear correlation. The stability of R^2^ values across different radii demonstrates the robustness of our image processing approach for dwell‐time verification.

These results confirm that the proposed image processing technique provides a reliable method for assessing dwell‐position accuracy and dwell‐time linearity in HDR brachytherapy QA procedures.

## 4 DISCUSSION

This study introduces a novel, automated methodology for HDR brachytherapy QA, integrating radiochromic film analysis with advanced image processing techniques to simultaneously and accurately evaluate source dwell position and dwell‐time linearity. Our approach achieves sub‐millimeter positional accuracy (≤0.6 mm) and exceptional temporal linearity (*R^2^≥*0.999) and was validated through a robust analytical framework. This automated system is designed to enhance treatment precision and streamline the clinical workflow. Additionally, it markedly reduces both manual intervention and staff radiation exposure, addressing key challenges in contemporary radiotherapy practice.

The clinical efficacy of HDR brachytherapy, particularly in image‐guided modalities (IGBT), is critically dependent on the precise spatial and temporal control of the radioactive source. As highlighted in ICRP reports 86 and 97, deviations in source trajectory or timing, whether from equipment malfunction or human error, can lead to severe clinical consequences.^22^, ^23^ Consequently, rigorous and periodic verification of these parameters is mandated. However, conventional clinical QA practices are often fraught with limitations. Dwell‐time verification frequently relies on manual stopwatch measurements synchronized with visual source confirmation, a process prone to human errors and unnecessary radiation exposure. Similarly, positional analysis based on the visual inspection of exposed films, even when aided by software such as RIT, is susceptible to significant inter‐observer variability and requires meticulous and time‐consuming film alignment.^17^

This study addresses the critical issue of film positioning errors through automated rotation correction. As shown in Figure 5(b), even when a film rotation error of 2° is artificially introduced, the analysis remains consistent with the non‐rotated case. The algorithm, combining Canny edge detection and HT line detection, accurately determines the rotation angle irrespective of the film edge slope, enabling precise correction via coordinate transformation. This effectively eliminates the influence of manual positioning errors on the final QA results, saving time and improving accuracy compared to that achieved using traditional methods.

Dwell‐time linearity is another cornerstone for ensuring the safety of HDR brachytherapy, as treatment planning systems often utilize optimization algorithms that may generate extremely short dwell times. In such cases, even minor timing inaccuracies can translate into significant dose deviations. Our methodology objectively quantifies linearity by correlating the programmed dwell time (*T_d_*) with the integrated pixel gray‐value sum (Σ) within a defined ROI, using the coefficient of determination (*R^2^*) as a direct measure of performance. Interestingly, our analysis revealed that increasing scanning resolution beyond 300 dpi (within the dpi range of 150–400) did not yield a more accurate or stable linear correlation. This is attributable to the physical nature of dose deposition, where radiation scatter creates a penumbra‐like effect at the edge of the exposure spots, rather than a sharp boundary. Consequently, higher resolutions capture this gradual transition without improving the integrity of the core signal, while unnecessarily increasing computational overhead. This finding suggests that 300 dpi is an optimal resolution for balancing accuracy and efficiency in this application.

In overcoming the limitations of traditional QA, our methodology offers several distinct advantages.

1. Enhanced Accuracy and Objectivity: By employing automated coordinate calibration and Gaussian fitting, the system achieves objective sub‐millimeter precision in localizing dwell positions, eliminating the subjective interpretation inherent in manual analysis and providing highly reliable and reproducible measurements.
2. Improved Safety and Robust Temporal Verification: The quantitative assessment of dwell‐time linearity through radial pixel summation obviates the need for manual timing, markedly mitigating radiation exposure risks for clinical staff. The high *R^2^* values achieved confirm the robustness of the method for temporal verification.
3. Optimized Workflow and Standardization: The fully automated pipeline, from scanned film import to final report generation, streamlines the entire QA process. This not only enhances operational efficiency but also promotes standardized, reproducible data reporting, aligning with the risk‐informed quality management principles advocated by AAPM Task Group 100.^24^

## 5 CONCLUSION

In conclusion, this study presents an innovative and automated analytical framework for assessing dwell‐position accuracy and dwell‐time linearity in HDR brachytherapy, leveraging radiochromic film dosimetry and advanced image processing techniques. The method achieves sub‐millimeter positional accuracy and excellent temporal linearity, as demonstrated through robust experimental validation. By eliminating manual intervention, enhancing workflow efficiency, and reducing radiation exposure risks, this approach not only ensures precise treatment delivery but also supports cost‐effective QA practices, ultimately advancing the safety and efficacy of precision radiotherapy.

## AUTHOR CONTRIBUTIONS

**Qingqing Yuan**: Data collection; drafting of the manuscript. **Suyan Bi**: Drafting of the manuscript; data analysis; final approval of the submitted version. **En Lou** and **YaJun Jia**: Data analysis. **Zhitao Dai**: Corrisponding author; final approval of the submitted version and funding support.

## CONFLICT OF INTEREST STATEMENT

The authors declare no conflicts of interest.

## CONSENT FOR PUBLICATION

We state that the consents for publication of data have been obtained from all patients.

## Data Availability

The datasets used and/or analyzed during the current study are available from the corresponding author on reasonable request.
